# Audit of Resuscitation Equipment in a Mental Health Setting – Resulting in Trust-Wide Action

**DOI:** 10.1192/bjo.2024.591

**Published:** 2024-08-01

**Authors:** Natasha Knowles, Noori Husain

**Affiliations:** Prospect Park Hospital, Berkshire, United Kingdom

## Abstract

**Aims:**

The aim of this audit was to review the availability of recommended resuscitation equipment in Prospect Park Hospital (PPH), a psychiatric hospital based in Berkshire.

The objective was to improve patient safety standards and address staff concerns by ensuring that recommended resuscitation equipment was accessible and fit for purpose.

Our hypothesis was that the current standard of resuscitation equipment at PPH was unsatisfactory.

Background

This review followed concerns by doctors who struggled to obtain the necessary equipment required for emergency situations, particularly during their out of hours shifts.

This project was significant as within the previous year there had been two incident reports and extensive anecdotal evidence of equipment failure/absence.

Whilst each ward had been tasked with completing a weekly checklist issued by the Resuscitation team, these had not been audited to ensure that standards were being met.

**Methods:**

Data was collected from ten locations at Prospect Park Hospital from 9^th^ May to 15^th^ May 2023.

Information was obtained by two doctors visiting the specified wards, reviewing the resuscitation bag equipment based on the standardised checklist.

The standards used were from local trust policy and Resuscitation Council UK policy.

**Results:**

7/10 locations did not meet the standards for resuscitation equipment, including missing or expired equipment such as adrenaline, suction devices and oxygen masks.

4/10 wards had not completed the weekly emergency drug checklist within the stipulated time frame.

70% of staff completed checklists were incorrect.

**Conclusion:**

Our hypothesis was proven to be correct, in that the current standard of resuscitation equipment at PPH was unsatisfactory.

We worked closely with the Resuscitation lead to recommend improvements, including an updated, more detailed checklist, a standardised procedure for ward managers and regular future audits.

Due to the significance of the findings, this has since been re-audited and is in the process of being rolled out Trust-wide, including all inpatient and community settings.

As a result of this audit, the Resuscitation team have been granted additional staffing to action these changes and increased their remit to monitoring equipment in addition to training.

These findings demonstrate that it is vital that the recommended resuscitation equipment is available and suitably maintained, particularly in a community hospital setting with limited resources where it can be life-saving.

